# Travelers’ attitude towards carpooling in Islamabad

**DOI:** 10.1186/s44147-021-00023-x

**Published:** 2021-11-05

**Authors:** Altaf Ayaz, Abdul Waheed, Hamza Saleem, Malik Muneeb Abid

**Affiliations:** 1 School of Civil and Environmental Engineering, National University of Sciences and Technology, Islamabad, 44000 Pakistan; 2 Department of Civil Engineering, College of Engineering and Technology, University of Sargodha, Sargodha, Pakistan

**Keywords:** Exploratory factor analysis, Questionnaire survey, Data validation, Structural equation model, Sample size, Carpooling, Confirmatory factor analysis

## Abstract

Islamabad, being the capital of Pakistan, is attracting every business. Thus, the city is growing towards traffic congestion as the city’s car ownership rate is rapidly growing. In such a situation, for successful implementation, the policymakers need to understand the public acceptance of carpooling services based on its key motives and constraints. This research explores the key motives and constraints to the introduction scenarios of carpooling service in Islamabad. A stated preference questionnaire survey was conducted via Google Form comprising several parts relating to carpooling. Exploratory and confirmatory factor analyses were processed, and a structural model was developed. Females (both single and married) were less orientated to carpool with males and married males with females. Unknown carpooling partners negatively influenced the factor of intention to shift to carpooling service. Our study provides policymakers and transport planners with an appropriate forecasting model of significant factors. In addition, it provides suggestions to transport planners to design promotional tools to enhance the tendency of carpooling among private car users in favor of reducing traffic congestion and increased car ownership rate in the city.

## Introduction

### Brief history

The term carpooling is widely used since the 1970s in response to the oil crises at that time. However, its history is still connecting to more back dates of time. In the early days of World War I when the USA met the economic declines, car owners started offering seats in their cars [1, 2]. This step got much success for short times, and the car manufacturers were affected very badly. So, they promoted their policies to a new and advanced level, which ultimately showed an almost 90% decline in carpools [3].

In today’s time, mobile applications operating through Internet connections promote carpooling [4]. Companies like Uber, Careem, Swvl, BlaBlaCar, Lyftshare, and Zimride are saturating the market day to day. Through these mobile applications, one can request a ride while sitting at his workplace. It provides the riders quiet ease in booking a ride, which makes it attractive once again. While dealing with these setups, the specific platform-based organizations are accelerating its rapid market spread, so meeting a massive range of clients to create their matches is conceivable [5].

### Different technical terms

Different authors have defined the term “carpool” differently. However, still, we can find agreements and correlations in their definitions. Let us discuss some of them.

#### “A setup in which more than one individuals share the use of a personally owned vehicle for a trip and the travelers pay to the driver’s trip-related costs” [6]

With the simplest approach, the carpool term is just a traveling method that needs at least one driver and one passenger who shares their rides from the same origin to the same destination. However, this situation becomes difficult to handle if the passenger needs to drop at some different locations. Another most complex form of this setup persists when the driver has to pick the passenger from some defined location and then again in the same trip needs to be dropped off at the originated location again. Such a situation leads the trips to indirect routes, longer distances, and increased travel time [7]. Therefore, the most indirect route trips are assumed to be made for far-away places to minimize the lost time. Some usual trip purposes of carpool are shopping, time-out activities, school, picking up children, and work. But the longest trips via carpool are generally found to be work-based [6].

#### In our everyday life, we use two terms “carpooling” and “carsharing” as in the same context, but they must be distinguished from each other

As per the definition of Millard-Ball et al. [8] “carsharing is hiring a vehicle for some time (in hours).” While comparing both modes, carsharing does not refer to the ownership of the car. In carsharing, the vehicle belongs to a company, and the users make the trips all alone in the hired car [9]. The fundamental carsharing goal is to reduce privately owned vehicles’ load for the infrastructure built without disturbing the accessibility factor of vehicles.

#### Another variation needs to be clarified between “on-demand” and “sharing” systems

Private systems like Uber, Careem, and Lyft deal in taxi-like facilities. However, the trip needs to be made by customers’ order via their online android and IOS applications. That is why such trips are mostly called ride-hailing rather than considering them as ridesharing [10]. So, one must be taking care of all of these terminologies while conducting any study.

#### Two more such terms “slugging” and “hitchhiking” refer to a person sitting at the roadside and waiting for a car to come and get him picked up to his destination location

The difference among them is that in carpooling, the passengers have to split the cost while slugging and hitchhiking do not involve splitting the costs. Another difference is that the term “slugging” is commonly used in US research while “hitchhiking” is commonly used in European papers. Both of these terms should not be used synonymously with carpooling even though both forms of traveling fits in the informal carpooling category [11].

#### Apart from the terminologies described above, “liftsharing,” “ridesharing,” and “motor-pooling” can be taken as the substitutes for the context of carpooling

But as per the literature and searches through Google Scholar, the common use of the term “carpooling” or “carpool” worldwide is followed by “carsharing.” Besides these searches, a literature also predicts the technicality of the term ridesharing that covers the models for traffic assignments, dynamic ridesharing algorithms, and optimization of ridesharing systems [12, 13]. Commonly, a mobile phone application is specified by ridesharing systems. The literature always addresses this type of carpooling as dynamic carpooling [11]. At the same time, the titles like liftsharing and motor sharing are rarely seen in the literature. The same system of vanpooling has also been introduced but just with a little difference in the larger vehicle size. Any company specially organizes vanpooling for their employees [14].

#### Ridesharing for more clarifications can further be studied from Roukouni and de Almeida Correia [15] and Chen and Shaheen studies [1]

Aiming at past studies or normal descriptions of all of the ridesharing terms discussed above, “an arrangement between two or more than two persons commuting together in the same vehicle having their destination locations on the same route and dividing the travel costs” is called carpooling.

**Fig. 1:**
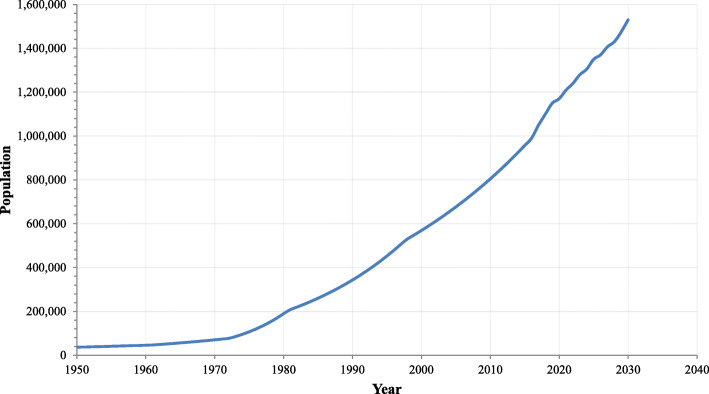
Islamabad population growth (source: World Population Review [17])

Traffic congestion due to the inclination in the car ownership rate has become a serious problem in today’s world [16]. Central business districts mostly meet the rise in vehicle ownerships because it attracts people due to the various facilities. Islamabad is also one of the same cases. Currently, traffic congestion is not a severely noticeable problem in Islamabad. However, the city will meet this issue in the future as the city’s population is rapidly growing every year with a growth rate of 3.05% [17] (Fig. 1) and so the auto ownership rate. Therefore, policymakers and transport planners need to consider strategic planning and transport studies for Islamabad in advance. Carpooling can reduce the auto ownership rate that can be proved as the most economical and feasible solution.

### Objectives

This study aims at the following objectives:

- To analyze the factors of traveler’s attitudes towards different carpooling scenarios
- Presenting key motives and constraints for the introduction scenarios of carpooling service within the city
- To develop a structural model for significant factors affecting travelers’ attitudes towards carpooling in Islamabad

## Literature review

In the current scenario of the transportation sector, some challenges like noise pollution and congestion are wasting our limited resources [18]. Mode shift towards public transports is not always found the best solution to such issues. Let us take an example of public transport; if the occupancy is low, the public transport will still not be sustainable [19]. So, to eliminate such problems, transportation planners and policymakers usually introduce a substitutional transport mode that can be “carpooling” to better the transportation operations [20].

Carpooling is a term used to specify the scenario of traveling in a single car by more than one person. Carpooling helps in reducing the number of vehicles of single occupancy [7]. It also allows travelers to get flexibility and low-cost trips. This traveling system is quite friendly to the environment, reducing emissions that keep society safe and natural [21]. As per the report of the US Environmental Protection Agency in 2005, travelers, especially the employees, can cash these benefits of carpooling, i.e., time-saving, lower costs. In carpooling, time utilization becomes greater than usual, i.e., time value for sleeping or reading purposes while traveling (because the rider does not drive). However, in the case of employers, parking lot demands reduce. Carpooling can use existing infrastructure, so public investments are also not required to construct separate traffic lanes [22].

The choice of carpooling of the trip makers depends on attitudes, demographic characters, and socioeconomic factors. A major study was conducted by Neoh et al. [7] in 2017, analyzing studies published till 2014, to find those factors that can influence the traveler’s decision of choosing carpooling as their trip mode. Their offered policies, rules, regulations, and facilities were also being analyzed to improve carpool involvement. This research categorized the key factors affecting carpooling into two main groups, i.e., “internal” and “external” factors. Internal factors include demographics and judgmental aspects while external factors include interventions and on spot situational aspects. As per the current situations, new technologies and modernizations are introduced into our society so the “sharing economy” idea is on the top priority [4]. Policies also need to be amended as per the current situations to meet the society demands and trends.

Seyedabrishami et al. [23] support the likely rise in the number of carpoolers using suitable strategies which indirectly will benefit the fuel consumption rate. Ridesharing in Canada and the USA represents almost 8 to 11% of the transport mode share. Also, there is a need to evaluate its association with traffic congestion, structure development, emissions, and energy consumption [1]. In a similar context, Manzini and Pareschi [24] support carpooling as an effective approach for the reduction of travel demands and costs. The accompanying issues such as travel demand management action acceptability by the people and its usefulness in varying travel attitudes need to be assessed. Carpooling policies for such aspects may be influenced by various factors: lifestyle, sociodemographics, and travelers’ intentions. Many researchers support the likely impact of individuals’ lifestyle and behavior upon travel-associated strategies [25–27].

Some other studies have also been contributed to the significant influence of various attitudes towards individuals’ travel behavior and travel demand management policies [28–30]. More specifically, a particular travel demand management policy is mainly succeeding due to the restrictions on personal vehicles and travel incentives [25]. Less cost, comfortable trip, and provision of well-kept facilities are found the main motives for a successful carpooling service as per the study of Sheldon and Heywood [31]. More travelers will carpool if the carpooling service was aided with high-occupancy vehicle lanes [32]. Seyedabrishami et al. [23] consider that high-occupancy vehicle lanes may not significantly affect travelers’ carpooling tendency, though it reduces the travel time for ridesharing. Agreeing to Li et al. [32], the main causes of carpooling are time-saving, liking excursions with others, and aiding the environment and culture. However, the program for matching the trip-mate, preferred parking at work, and owners’ carpooling incentives are the least significant aspects of carpooling for trips [32].

A study by Minett [33] concluded that a sufficient portion of bike and car drivers look for an alternative mode to commute, which offers equal comfort and less cost, as they feel too tired while driving alone. Some researchers believe that money saving is the key motive for carpooling, and thus, it is a mode of travel for low-income people (Correia and Viegas [34]). A recommendation of a proper setup for individuals’ trust and interaction with other travelers was also provided in their study. According to Ciari [35], safety, alone driving, guaranteed service for back home trips, and particular traveler’s group are the key motives for choosing carpooling. Moreover, travelers’ attitude, incentives on carpooling like money-saving, time and safety, purpose of the trip, trip regularity, age, and disincentives on personal vehicles may also be included [36, 37].

Malodia and Singla [38] indicated additional travel time, walk and waiting time on pick-up location, and cost saving as the influencing factors regarding carpooling utility for Indian cities. Travelers’ tendency for carpooling is significantly affected by the distance to the meeting site, parking cost, partner matching, and flexibility of services as per the result of a created probit model [39]. To encourage carpooling, an online survey was conducted at Michigan State University, and the same issue of finding carpool partners was identified [40]. Ostrovsky [41] also reported the socially optimal matching of the riders as the main outcome while studying the economical interactions among autonomous transport, carpooling, and road pricing. Srivastava [42] provided various factors like incentives, low cost, mixed approach, and incentives to drivers to improve such shared services use. Important factors found for carpooling are gender, nearness to car regions, encouragement to save time, and present transit services use (Soltys [43]).

Watts [44] indicated many constraints to carpooling such as household size, car ownership’s rate, variations in travel conduct, and attitudes. This study also supports the increment of vehicle occupancy rate for work purposes trips because of carpooling. Unlikely Asian countries, Delhomme and Gheorghiu [9] found women and people with children as more probable to be a carpooler. It was also found that the travelers with a positive approach towards public transport and aware of the environmental concerns are also more likely to be a carpooler [9].

Chen et al. [45] studied the important contributors to carpool by collecting data from three different cities of the USA with varying attributes and identified that promotion of carpool was significantly affected by the incentives for individual level; however, statistically significant differences were observed in carpool users for each city. Moreover, household lifestyle, parking finding time at work, gender, and age were marked as positively effecting factors for carpooling [45].

Data from the transportation survey in “The Ohio State University” was used to analyze the factors affecting carpooling preference and identified the positive association of travel distance, marital status, current mode of transportation, and attitudes towards carpooling [20]. The study also resulted in the variation of the motives and constraints for carpooling by role (present carpool users, expected carpool users, and not involved travelers). Mou et al. [46] conducted an online survey in Jinan, China, to examine the effect of carpooling on car purchasers’ buying intention and tested the proposed hypothesis using the structural equation modeling technique. They found about 22% of car purchasers delayed buying a car while 12% of them even changed their decision of purchasing a car [46].

Ordinary least squares approach was used to analyze the behavior of carpoolers in traveling and resulted in a non-habitual aspect of carpooling for workers [47]. Sociodemographic characteristics like household composition, age, gender, and being native were indicated in a constant correlation with carpooling partaking [47]. Moreover, it is stated that the approach towards carpooling may get declined due to the current COVID-19 pandemic [47]. An attempt to map commuters’ attitude (cognitive complexity and empowerment perceptions) towards carpooling services has been made. A scenario-based survey was conducted from the carpooling experienced persons. MANOVA analysis has been used which showed the significant influence of both the attitudes on the motivational constructs [48].

The worldwide studied literature delivers many of the factors that positively contribute to the carpooling policies. However, there are certain variables that negatively influence this service. Still it is essential to classify the possibility of such travel demand management strategies in the local socioeconomic perspective of Islamabad. This is very much important to study because the characteristics of the travelers change with respect to the city or a country. People of each city have their own unique lifestyles with socioeconomic characteristics regarding culture, population growth, transportation problems and its infrastructure, and land use pattern. In such situation, the imposition of procedures established on the judgments for other cities may poorly affect the whole transportation system of the policy-implementing city and which may cause the poor acceptability of the public towards it. Thus, it should be considered as a foremost priority for a vital study of the local factors of the targeted city Islamabad to introduce some new transportation policies.

## Methods

**Fig. 2:**
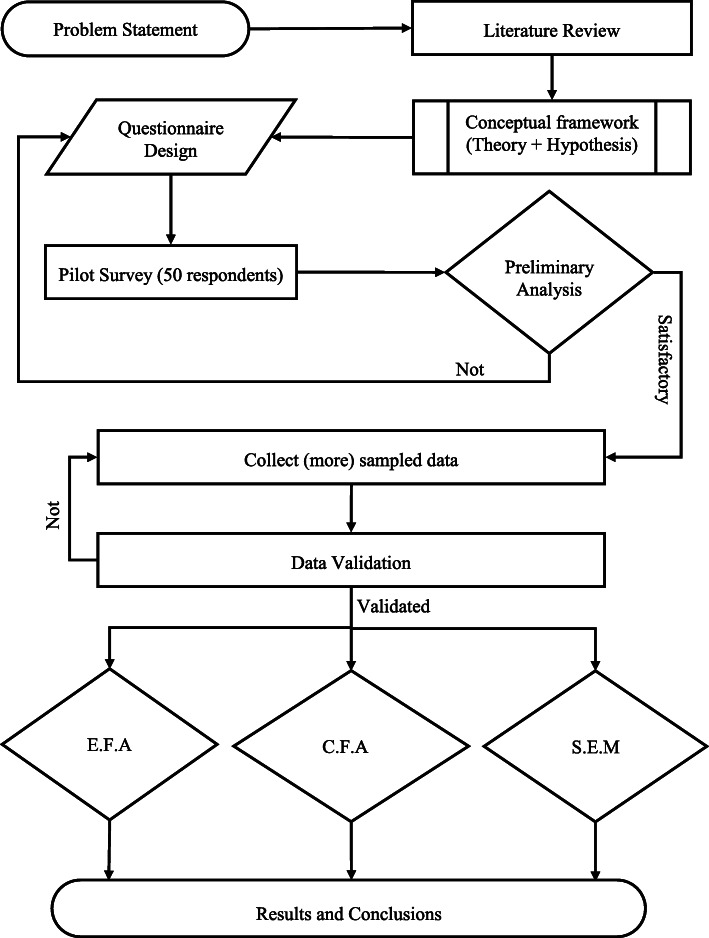
Research methodology flow diagram

A complete framework of the adopted methodology for this study is shown in Fig. 2. The stepwise description of which is discussed below.

### Study area

**Fig. 3:**
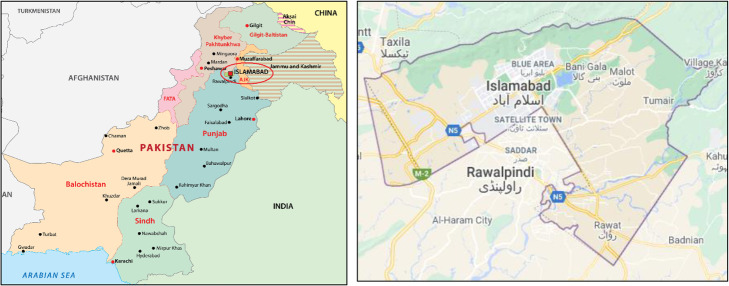
Islamabad city (Source: Google map)

Islamabad being the capital of Pakistan is attracting every business hence becoming a private car-dominating city and thus chosen for this study. The exact location of the city shown in Fig. 3 can be accessed with the coordinates 33.7205° N, 73.0405° E on Google Map.

### Hypothesis

**Fig. 4:**
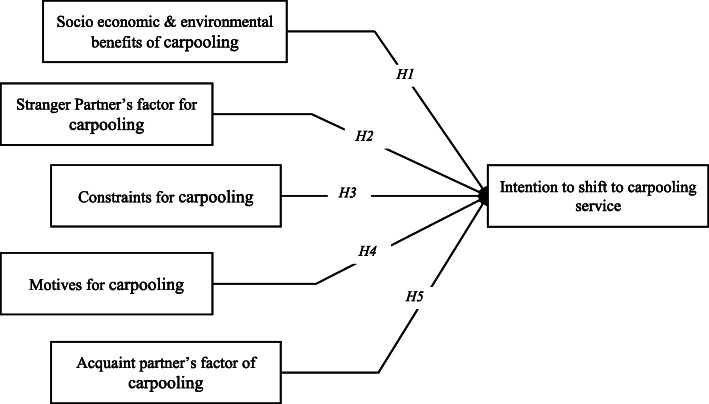
Conceptual model and hypothesis

Following the research trends of the literature related to the context of carpooling, our study also depends upon some theoretical assumptions. Five latent variables are taken from the literature for this study (see Fig. 4)

.

Ashraf et al. [49] and Sheldon and Heywood [31] concluded the significant impact of socioeconomic and environmental benefits of carpooling service on user’s tendency for using this service. Further, Ashraf et al. [49] reported motives for carpooling services such as cost reduction, free parking, and preference of traveling in high-occupancy vehicle lanes as positive factors for carpooling services. Similarly, constraints in preferring carpooling service such as privacy concerns, unknown passenger fellow, and sharing contact details were found in negative relation to carpooling service. de Almeida Correia et al. [28] found the problem of “stranger partner” variable in inverse relation to carpooling service and difficult to overcome. On the other hand, partners’ acquaintanceship factor for carpooling service is reported as positively affecting users’ intention to use carpooling service. Relying on the literature of these researches, all of these variables are considered for our study as well. The overall theoretical framework of the assumed hypothesis for this study is as defined as follows:

- *Hypothesis 1 (H1). Socioeconomic and environmental benefits of* carpooling *have a significant and positive effect on the intention to shift to* carpooling service*.*
- *Hypothesis 2 (H2). Stranger partner’s factors of* carpooling *have a significant and negative effect on the intention to shift to* carpooling service*.*
- *Hypothesis 3 (H3). Constraints for* carpooling *have a significant and negative effect on the intention to shift to* carpooling service*.*
- *Hypothesis 4 (H4). Factors of motives for* carpooling *have a significant and positive effect on the intention to shift to* carpooling service*.*
- *Hypothesis 5 (H5). Partners’ acquaintanceship in* carpooling *has a significant and positive effect on the intention to shift to* carpooling service*.*

### Questionnaire design and pilot survey

Questionnaire designed comprised five parts: (1) respondents’ personal and travel information, (2) travelers’ response to presented carpooling situations, (3) benefits of carpooling, (4) probability of switch to carpooling service and favoring own vehicle over carpooling, and (5) travelers’ response to carpooling with known and stranger partners. Items in the questionnaire were critically designed according to the hypothesis of the study and understanding of the respondents. The first part was used to distribute the respondents according to their social and demographic characteristics. The results of which are presented in Table 2 and Fig. 7. All other remaining sections of the questionnaire were measured on a Likert scale from 1 to 5: 1 being strongly disagreed and 5 strongly agreed with the presented scenario of the carpooling service within the questionnaire. The questionnaire was prepared on Google Form to collect data from the respondents online. Meanwhile, interviews were also conducted in the study area.

The targeted people were working or residing population of Islamabad city only, both potential and personal car users. An initial survey of 50 samples was conducted to check the accuracy of the items of the designed questionnaire. The questionnaire was improved as per outcomes of the initial survey of which the finalized version is as provided in Table 1.

**Table 1:** Coding and descriptions of the items in the questionnaire measured on a Likert scale

Construct	Coding	Variable description	Source
**Motives for carpooling**	M1	I will prefer carpooling service for short trips (within Islamabad).	
	M2	I will prefer carpooling service for long trips (outside Islamabad).	
	M3	I will prefer carpooling service shared with 1–2 persons.	[49]
	M4	I will prefer carpooling service shared with 3–4 persons.	[49]
	M5	I will prefer carpooling service if 25–50% cost reduction occurs.	[49]
	M6	I will prefer carpooling service if more than 50% cost reduction occurs.	[49]
	M7	I will prefer carpooling service with persons of opposite gender.	[50]
**Socioeconomic and environmental benefits**	B1	Carpooling can save my money for fueling.	[49]
	B2	Carpooling can save travel time if H.O.V lanes are provided.	[32]
	B3	Carpooling can reduce impacts on the environment.	[32]
	B4	Carpooling can reduce traffic congestion.	[51]
	B5	Carpooling can reduce energy consumption.	[49]
	B6	Carpooling can reduce stress for self-driving and keeping personal cars.	
	B7	Carpooling can reduce maintenance costs of personal cars.	[52]
**Intentions to shift to carpooling service**	**I will shift to carpooling service.**		
	I1	If carpooling costs are lower.	
	I2	Because I feel safe with another person driving.	[52]
	I3	If H.O.V lanes are provided.	[49]
	I4	If preference is given to pooled vehicles in parking lots.	[32]
	I5	If I get free parking.	[49]
	I6	If carpooling service is more comfortable.	[51]
**Constraints for carpooling**	**I will prefer to use my own car.**		
	C1	Because not knowing who my fellow passenger might be.	[49]
	C2	Because I do not want to depend on another person.	[28]
	C3	Because I do not want to give out my phone number to an unknown person.	[49]
	C4	Because of privacy.	[49]
	C5	Because carpooling can restrict my freedom.	[49]
**Partner’s acquaintance**	Aq1	Carpooling with one known man.	[28]
	Aq2	Carpooling with one known woman.	[28]
	Aq3	Carpooling with two known persons.	[28]
	Aq4	Carpooling with three known persons.	[28]
**Stranger partners**	S1	Carpooling with one unknown man.	[28]
	S2	Carpooling with one unknown woman.	[28]
	S3	Carpooling with two unknown persons.	[28]
	S4	Carpooling with three unknown persons.	[28]
	S5	Carpooling with one known and one unknown person.	[28]
	S6	Carpooling with one known and two unknown persons.	[28]

All these parameters are measured on a Likert scale from 1 to 5, i.e., 1, strongly disagree; 2, disagree; 3, neutral; 4, agree; 5, strongly agree

### Sample size calculation

After the questionnaire items were confirmed, a complete set of the calculated size of 600 samples was then collected using the Google form link. The sample size was calculated using previous studies’ approach [53, 54] at 95% confidence level, 4% margin of error, 50% male population, *Z*-score of 1.96 and 1.2M population size [17].

$$ \mathrm{Sample}\ \mathrm{size}=\left(\frac{\frac{Z^2\times p\ \left(1-p\right)}{e^2}}{1+\left(\kern0.5em \frac{Z^2\times p\ \left(1-p\right)}{e^2\ N}\kern0.5em \right)}\right)=\left(\frac{\frac{(1.96)^2\times 0.5\ \left(1-0.5\right)}{(0.04)^2}}{1+\left(\kern0.5em \frac{(1.96)^2\times 0.5\ \left(1-0.5\right)}{(0.04)^2\ (1200000)}\kern0.5em \right)}\right)=600\ \mathrm{samples} $$

Data screening was implied to exclude the unengaged, outlying, and missing responses [55]. The deficiency in the sample size, due to data screening, was completed by more fresh responses.

### Respondent’s profile

A total sample size of 600 responses was collected. Table 2 represents the respondents’ sociodemographic information. Females (23.2%) as compared to males (76.8%) are less because of religious, social, and cultural constraints in the study area.

**Table 2:** Respondents’ sociodemographic information

Variable	Distribution (%)
Gender	Male (76.8), female (23.2)
Marital status	Single (82.1), married (17.9)
Education	Matric/F.Sc/diploma (8.9), undergraduate (40.2), postgraduate (50.8)
Profession	Student (57.7), employee (28.0), business (4.9), others (9.3)
Age (years)	≤ 18 (6.1), 19–25 (72.0), 26–40 (20.7), ≥ 40 (1.2)
Income (PKR)	0–25k (60.2), 25k–50k (23.2), 50k–75k (4.5), 75k–100k (6.9), > 100k (5.3)
Current trip mode	Personal car (21.1), bike (27.6), metro bus (8.5), other public transport (26.4), office/university transport (8.9), pick and drop (2.4), others (4.9)
Current trip purpose	Education (48.8), work (43.5), others (7.7)
Vehicle ownership	Car (24.8), bike (28.5), both (7.7), none (39)
Driving license	Yes (48.8), no (51.2)
Awareness about carpooling	Not at all (29.7), slightly (28.9), somewhat (17.1), moderately (13.9), extremely (10.5)

Likewise, due to the social restrictions, very fewer responses from married people (17.9%) were received. A rich proportion of the respondents were in the age range of 19–25 years that are adults (72%), and so most of them were students (57.7%) followed by the proportion of employees (28%). Non-vehicle owners were 39%, while 24.8% car and 28.5% bike owners in the responses.

## Results and discussions

For descriptive analysis of the data, Microsoft Excel 2016 and SPSS are used. SPSS is a set of programming items and associated services for statistical analysis, presented by the International Business Machines Corporation IBM [56]. Analysis of moment structures (AMOS) is also being used for CFA and SEM [57] analysis. Microsoft Excel 2016 and Stat tool package by James Gaskin [58] were utilized for data screening and validity checks, respectively.

### Descriptive analysis

**Fig. 5:**
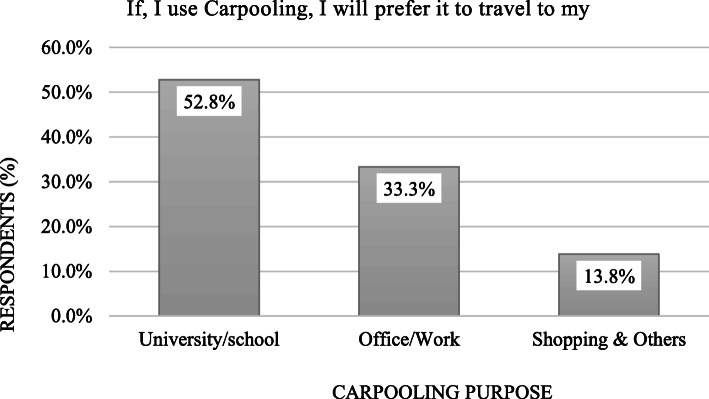
Respondents’ carpooling trip purpose

Figure 5 shows the response of the targeted population against the purposes of using carpooling in their case. Most of the surveyed respondents (52.8%) oriented towards using carpooling service for their educational (university/school) purposes followed by office/work trips (33.3%). This means that carpooling will get more attraction for people like students. This finding is similar to the past research [49]. Considering shopping or other carpooling trip purposes was found with the least response (13.8%).

**Fig. 6:**
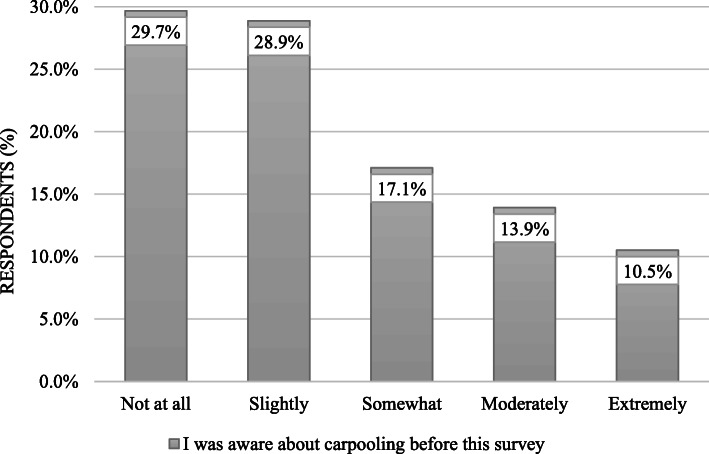
Awareness of respondents about carpooling before this study

At the start of the scenarios presented for carpooling, the respondents were asked for their past awareness about the carpooling system, and the results are as shown in Fig. 6, which shows 58.6% of respondents were either completely (29.7%) or slightly (28.9%) unaware of this service before this study. It is evident from Fig. 6 that the responses are positively skewed towards the origin, which clearly reports the unawareness of the people with this service before this study. To counter this issue in our study, some pictorial representations of the carpooling service were also provided with the questionnaire.

**Fig. 7:**
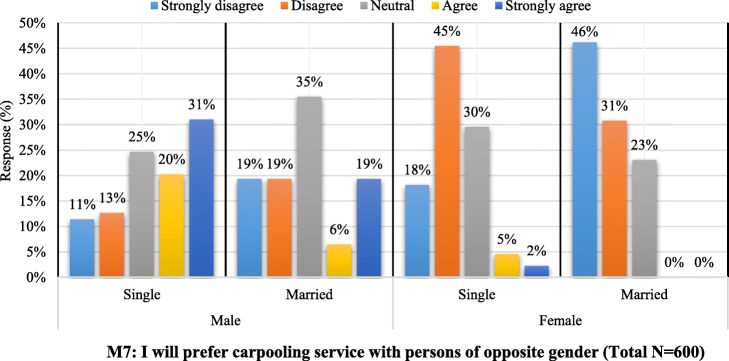
Willingness of respondents to travel with opposite gender partners (M7) in accordance with their marital status

One of the interesting findings of our study is provided in Fig. 7. This graph describes the attitude of the respondents towards carpooling with opposite gender partner based on their own gender and marital status. It is clearly observable that the graphs for the females, either single or married, are highly skewed positive, which indicates the non-agreeableness of females towards sharing their rides with males. Similarly, the data for married males is positively skewed which also reports their noticeable disagreeableness towards carpooling with females. These results are evident and reality predicting while considering the social and cultural influences of the people of Islamabad.

On the other hand, if we look at the graphical representation of the single males’ data, their result is skewed negatively (away from the origin), which predicts that unmarried males are more oriented towards carpooling with female partners. This is acceptable because the maximum responses received for this study were from the students, i.e., adults in the age range 19 to 25 years.

**Fig. 8:**
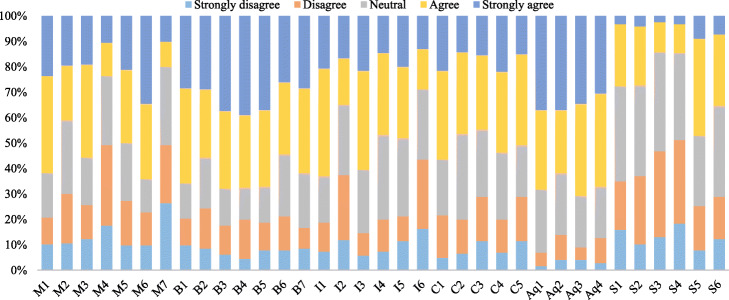
Responses to all the Likert scale variables taken for the study

If we consider the variable M7 separately as a whole, then we find that its skewness is positive with a value of 0.417 (Appendix). Figure 8 shows the response summary for all the items taken for the study on a Likert scale. It is quite noticeable that the responses for all the items are all sequential (almost the same pattern) except for the items relating to a strangers’ partner matching in carpooling (S1 to S6). This is the same as we expected in our hypothesis 2 and is in correspondence to the study of de Almeida Correia et al. [28]. Most of the travelers avoid (disagreed) traveling with stranger partners. Meanwhile, the result of the items for acquaintanceship of the carpooling partner is supportive to our hypothesis 5, i.e., greater agreeableness proportion at top of the graph.

**Fig. 9:**
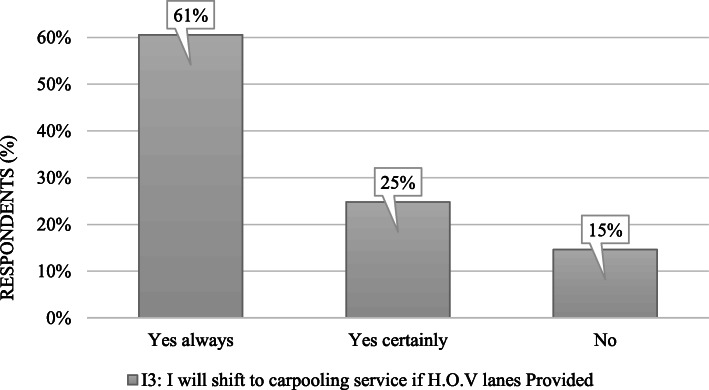
Intention to shift to carpooling, if high occupancy vehicle lanes provided

Furthermore, It can also be seen that the items (C1 to C5) representing constraint variables for carpooling are non-supportive to our hypothesis 3, i.e., higher ratio at the top of the graph. Finally, item I3 (*I will shift to carpooling service if high occupancy vehicle lanes are provided*) was summed up in a simple representation (see Fig. 9), which displays the merged responses of item I3, i.e., merging responses of “strongly agree” and “agree” to “yes always,” “neutral” to “yes certainly,” and “strongly disagree” and “disagree” to “no.” Figure 9 depicts that most of the travelers (61%) are willing to shift to carpooling service if high-occupancy vehicle lanes are provided. This result is similar to the study of Ashraf et al. [49].

### Model development

#### Reliability analysis

Reliability analysis indicates the way that a scale must reliably reveal the construct it is estimating [59]. Cronbach (1951) presented an approach that is normal in reliability analysis. This action, by any means, is generously identical to the separation of the data in two splits and further recording the correlation estimates respectively. Cronbach’s alpha is the average of these estimates which is tested to analyze the consistency of the factors in the designed questionnaire [60]. Its value must be greater than 0.7 in order to get good and acceptable reliability [61]. Table 3 represents the reliability analysis and is all greater than the standard value of 0.70, presenting a good internal consistent reliability among the variables. Appendix represents further descriptive statistics such as the mean, median, and mode for the items of these constructs. Thirty-five items were measured on a scale of 1 to 5. Their mean values range from 2.48 to 3.97 and similarly the standard deviations from 0.94 to 1.33.

**Table 3:** Cronbach’s alpha values obtained from reliability analysis

S.No	Construct	Acceptable ranges [61]	Cronbach’s alpha	Remarks
1	Socioeconomic and environmental benefits	> 0.9—excellent > 0.8—good > 0.7—acceptable > 0.6—questionable > 0.5—poor < 0.5—unacceptable	0.944	Excellent
2	Stranger partner		0.816	Good
3	Constraints for carpooling		0.873	Good
4	Partner’s acquaintance		0.904	Excellent
5	Motives for carpooling		0.845	Good
6	Intention to shift to carpooling service		0.761	Acceptable

#### Validity analysis

Validity analysis is a fundamental portion of empirical study. Typically, in case of a questionnaire, content and structure validity is selected for measuring. Content validity indicates the suitability and normal consistency among the objects and the tested variables. It actually answers whether the test is fully representative of what it targets to measure [62]. This study focuses on the structure validity, rather than the content validity, as the questionnaire items used in this research are established from the literature review. Structural validation alludes to the ability of objects of estimating the variables. It refers to the ability of the test to measure the concept that it wished for [62]. EFA was conducted to test the structural validity of the variables. The convergent and discriminant validity of the variables are presented in Table 4. The values of composite reliability for the latent variables are all greater than 0.6 (see Table 4), which reports the good model reliability [63]. Similarly, the values of average variance extracted for all the constructs are > 0.5, confirming the convergent validity of the model [64].

**Table 4:** Test results of convergent and discriminant validity

	CR	AVE	Intentions	Benefits	Stranger	Constraints	Acquaint	Motives
**Intentions**	0.803	0.580	**0.77**					
**Benefits**	0.941	0.698	0.54	**0.84**				
**Stranger**	0.878	0.598	0.26	0.15	**0.77**			
**Constraints**	0.858	0.550	0.31	0.34	− 0.07	**0.74**		
**Acquaint**	0.906	0.707	0.24	0.45	0.3	0.14	**0.84**	
**Motives**	0.825	0.503	0.43	0.60	0.43	0.15	0.36	**0.71**

*CR* composite reliability, *AVE* average variance extracted

Table 3 is obtained by using the master validity sheet in stat tools package prepared by Gaskin [65] in which the diagonal values are obtained from square rooting the value of AVE in Table 4 for each construct, which are all higher than the inter-construct correlations (off-diagonal values), thereby confirming discriminant validity for the model [64]. The covariance of motives with benefits is high (0.60), but still, less than the diagonal value of 0.71 thus fulfilled the validation.

#### KMO and Bartlett’s test

The calculated and collected sample size of 600 responses was checked by KMO and Bartlett’s test with KMO measure 0.837 (> 0.70) which is great [66], and Bartlett’s test is remarkable, i.e., significant at 0.000 < 0.005. These outcomes showed that the data were reliable with the requisite of EFA. Thus, further analysis was proceeded by utilizing principal component analysis in factor analysis under dimension reduction in SPSS.

#### Exploratory factor analysis (EFA) by factors rotation

Factor rotation for exploratory factor analysis (EFA) was performed, and a number of iterations were checked in IBM SPSS until a clear pattern matrix was obtained, based on which a confirmatory factor analysis (CFA) was also implied and a structural equation model (SEM) was developed using IBM SPSS AMOS. Promax oblique rotation was utilized for EFA with the options of “sorted by size” and “suppress small coefficients” under the value 0.5. PCA was performed in SPSS 23.0, and the outcomes are as shown in Table 5, presenting the high factor loading on each item except I2 (0.524). Still, the overall loading on each construct is too good as the mean values are all greater than 0.70 [67]. Further, the communalities are all pretty higher than 0.3, so the results can be processed further with CFA. Cumulative variance explained 71.675% is quite good [67].

**Table 5:** PCA pattern matrix and exploratory factor analysis (EFA)

Latent construct	Items	Factor loading	Loadings mean	Variance explained (%)	Communalities
**Socioeconomic and environmental benefits**	B3	.968	.843	32.551	0.853
	B4	.933			0.834
	B1	.903			0.836
	B5	.872			0.846
	B6	.785			0.696
	B7	.727			0.701
	B2	.711			0.617
**Constraints for carpooling**	C5	.901	.803	12.486	0.757
	C4	.857			0.752
	C2	.837			0.724
	C1	.749			0.66
	C3	.670			0.636
**Partner’s acquaintance**	Aq3	.928	.875	9.100	0.868
	Aq4	.888			0.794
	Aq1	.848			0.765
	Aq2	.834			0.694
**Intention to shift to carpooling service**	I4	.853	.724	8.245	0.732
	I5	.766			0.69
	I6	.754			0.577
	I2	.524			0.409
**Stranger partner**	S4	.878	.832	5.258	0.704
	S2	.817			0.794
	S1	.801			0.759
**Motives for carpooling**	M7	.806	.713	4.036	0.697
	M4	.735			0.594
	M2	.681			0.662
	M5	.631			0.701
Cumulative variance explained				71.675%	

EFA is an iterative process that helps in assembling multiple observed variables into few latent constructs and drops off the irrelevant or insignificant variables from the study. Relying on the results of EFA, this study also dropped one latent construct of “awareness about carpooling” after the pilot survey, as it was not providing some reliable and significant results and the clear pattern matrix was unable to obtain in the EFA. Looking at the clean pattern matrix in Table 5 with no cross-loadings, we can conclude that the factors relating to the socioeconomic and environmental benefits of carpooling service have the greatest tendency for a traveler to use this mode as their factor loadings are comparatively quite higher and similarly the partner’s acquaintanceship factor as well. Surprisingly, motives have the least loadings, which is a little contradictory to the reality, but can be improved in future researches by adding other suitable variables or subtracting any of the limited variables in our study. At the end, as expected, stranger partner variable is obtained with the lower factor loadings for the travelers to use carpooling service as their transport mode.

#### Confirmatory factor analysis (CFA)

There are several goodness of fit indices for a CFA model to evaluate. The insignificant chi-square test with *p* > 0.05 refers to a good fit of the model. This test is too responsive to large sample sizes. Thus, to minimize the impact of sample size, an alternative normed chi-square (Σ2/df) approach is used, whose value is taken acceptable fit from 2 to 5 [68]. Some other indices like root mean square error of approximation (RMSEA) with a recommended value ≤ 0.06, comparative fit index (CFI) with a recommended value ≥ 0.95, standardized root mean square residual (SRMR) with a recommended value ≤ 0.08, and Tucker-Lewis index (TLI) with a recommended value ≥ 0.95 indicate an excellent model fit [68].

In Table 6, five model fit indices were checked for the measured model, only one out of which did not meet the excellent model fit levels but still good enough to be considered. The remaining four indices are pretty high and acceptable as satisfying the excellency standards, thereby as a whole indicating that the model fits the data quite well.

**Table 6:** Model fit results and acceptable thresholds of the model fit indices

Goodness of fit indices	Excellency level [68]	Measured model value	Remarks
Chi-square/df	< 5	3.317	Acceptable
CFI	≥ 0.95	0.952	Acceptable
TLI	≥ 0.95	0.961	Acceptable
RMSEA	< 0.06	0.068	Questionable
SRMR	< 0.08	0.073	Acceptable

*CFI* Comparative fit index, *TLI* Tucker-Lewis index, *RMSEA* root mean square error of approximation, *SRMR* standardized root mean square residual

The loading of observed variables on latent constructs in Table 5 is all high enough to accept, as their mean values are all greater than 0.6. The covariance of “benefits of using carpooling service” with the “motives to use carpooling service” in Table 4 was alarming with a value of 0.6. Normally, we do not want these covariances to have high values as they may raise validity concerns. In our case, it is still less than the diagonal value of motives (0.71) and benefits (0.84) in Table 4, so it is accepted.

#### Structural equation model (SEM)

SEM has always been used extensively in the literature for behavioral studies [49, 68, 69]. Socioeconomic and environmental benefits of carpooling service—H1—positively affected the intention to use carpooling service (*β* = 0.38, *р* < 0.05). This result is similar to that of Ashraf et al. [49] indicating that the best carpooling service would be the one providing the most possible socioeconomic and environmental benefits to the travelers. Stranger partner’s factor—H2, where the partners in a pooled car do not know each other—negatively affected the intention to use carpooling service (*β* = − 0.16, *р* < 0.05). This is the same as we expected in our hypothesis and is obvious to human nature as well. de Almeida Correia et al. [28] also reported a similar result and found that this situation is not easy to overcome. Constraints for carpooling—H3—had a non-significant and positive effect on the intention to shift to carpooling service. Our analysis in the structural model did not support hypothesis 3 (*β* = 0.21, *р* > 0.05). This means that the users’ intention to use carpooling services is not affected by the constraints of preferring privately owned cars (see Table 1 for the description of variables). This additionally conveys the conclusion that the car users are also willing to shift to carpooling services if found to be more attractive in the context of cost or convenience. This outcome is quite interesting and different from our expectations but favorable to the introduction of carpooling services. Motives for carpooling—H4 (*β* = 0.25, *р* < 0.05)—and partners’ acquaintanceship—H5 (*β* = 0.32, *р* < 0.05)—both positively and significantly affected the factor of intention to use carpooling services.

The results are similar to Ashraf et al. [49] and de Almeida Correia et al. [28]. Ashraf et al. [49] studied the motives for carpooling for a very similar study area as ours. The behavior of the people is almost the same in both areas. The similarity of the results is showing high reliability of the variables and methodology used. de Almeida Correia et al. [28] conducted a study on carpooling using the SEM technique and found that the partner acquaintanceship is highly relatable to the choice of carpooling. Different types of iterations were made, and a positive impact of acquaintance and a negative impact of stranger variables were concluded respectively.

**Fig. 10:**
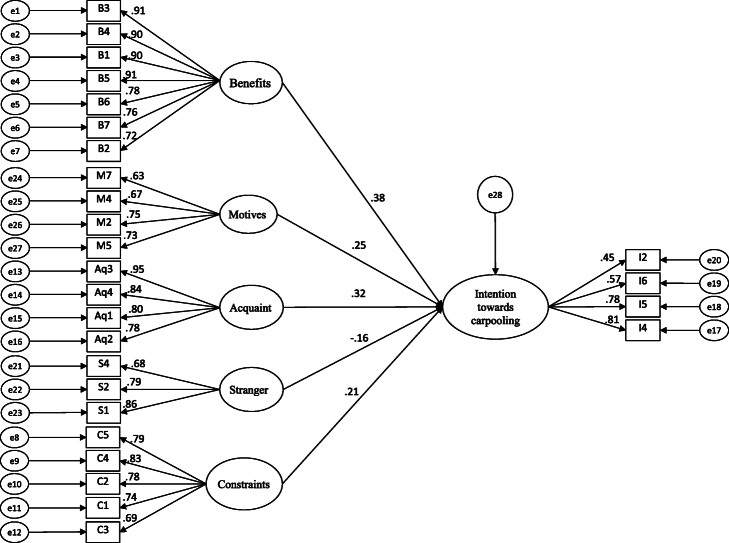
Structural model

Altogether, our research model explained 71.6% of the variance in intention to shift to carpooling services (see Table 5). The results of the standardized estimates of the path analysis are shown in Table 7 and Fig. 10. Figure 10 reports only the significant items, while the insignificant items were excluded from the model. Similar to CFA, the structural model has also been checked for model fit with the same indices. The results of the goodness of fit indices are as follows: Σ2/df = 3.317, CFI = 0.936, TLI = 0.961, RMSEA = 0.058, and SRMR = 0.091, confirming good model fit.

**Table 7:** Results of structural model

Path			Hypothesis	Estimate	S.E.	*p*-value	Remarks
Intention to shift to carpooling service	←	Benefit	H1	0.38	0.052	0.001	Supported
	←	Stranger	H2	− 0.16	0.060	0.000	Supported
	←	Constraints	H3	0.21	0.087	0.530	Not supported
	←	Motives	H4	0.25	0.081	0.017	Supported
	←	Acquaint	H5	0.32	0.055	0.022	Supported

In the developed model of this study, socioeconomic and environmental benefits of carpooling services have the highest probability of 38% followed by partner acquaintanceship factor (32%) and motives for carpooling, i.e., 25% effect on travelers’ choice to use carpooling as their mode of transportation. This study concludes these latent constructs are the key motives for carpooling services. On the other hand, stranger partner is negatively influencing the intention to shift to carpooling service, i.e., −16%, which represents this variable as the only constraint for carpooling service identified by this study. The other latent construct of “constraint” was found insignificant (*p* > 0.05) in the model.

## Limitation

All studies report some limitations. Acknowledgment of a study’s limitations is an opportunity to make suggestions for further researches. Following the same trend, our study also reports some limitations as indicated below.

The collected sample size is small which may differ from the results if conducted with a greater sample size of more than 1000. Still, our findings are valid and applicable and can be used as a baseline for future studies. Furthermore, an extension to this research can be provided in future studies by taking new and suitable variables.

## Conclusions

Regardless of the limitation discussed above, this research makes novel addition to the current pool of studies concerning carpooling. The outcomes of this study affirmed the validity of the models concerning public acceptance towards carpooling. Car users’ intention to use carpooling services is not affected by the constraints of preferring privately owned car (H3—not supported). One of the interesting findings of this study is the non-agreeableness of females (both single and married) towards sharing their rides with males and similarly the case of married males with females. On the other hand, unmarried males are more oriented towards carpooling with female partners. Similar partner matching has always been an issue in carpooling systems. Meanwhile, the model in this study also supports the negative effect of an unknown partner on the intention to use carpooling service. Carpooling service can attract sufficient travelers (61%) if high-occupancy vehicle lanes are provided. Understanding people’s acceptance of carpooling scenarios reveals the importance of the plan and assists us with planning methods for successfully implementing the carpooling system in the future.

Many proposals can be provided to policy-makers, i.e.:

- Considering the results of Fig. 6, carpooling use can be encouraged by informing the travelers about carpooling benefits that are reported in this study and by many other researches, i.e., Shaheen et al. [70]
- Government support is required for the efficient introduction of high-occupancy vehicle lanes for carpooling service in the city as most of the travelers (61%) are willing to shift to carpooling service if high-occupancy vehicle lanes are provided.
- Based on the results of Fig. 7, market promotion of carpooling should consider the gender and marital status of the travelers.
- Various schemes can be incorporated in carpooling services (e.g., free parking lots and prioritizing high occupancy vehicle lanes) to build the practicality of carpooling services for decreasing car ownerships.
- Concurrently, the local government ought to create strategies to control the increasing car ownership rate (e.g., imposing parking fees on personal car use, car use restrictions, and low emission zones).
- Overall, through assessing the impacts of attitudinal elements on carpooling selection in a capital city like Islamabad, this study can give directions to other large cities in developing countries.

## Data Availability

All the relevant data has been included in the article.

## Appendix

**Table 8:** Descriptive statistics of the items taken for EFA, CFA, and SEM

Variable	*N*, valid	Mean	Median	Mode	Std. deviation	Variance	Skewness	Kurtosis
M1	600	3.54	4.00	4	1.244	1.547	− .700	− .478
M2	600	3.20	3.00	3	1.257	1.581	− .108	− .971
M3	600	3.37	4.00	4	1.273	1.622	− .519	− .796
M4	600	2.67	3.00	2	1.212	1.469	.410	− .685
M5	600	3.34	3.50	4	1.261	1.589	− .322	− .936
M6	600	3.66	4.00	5	1.329	1.767	− .706	− .721
M7	600	2.54	3.00	3	1.260	1.588	.417	− .723
B1	600	3.64	4.00	4	1.266	1.602	− .786	− .423
B2	600	3.52	4.00	5	1.290	1.663	− .456	− .921
B3	600	3.82	4.00	5	1.224	1.497	− .832	− .338
B4	600	3.82	4.00	5	1.229	1.511	− .745	− .628
B5	600	3.78	4.00	5	1.266	1.603	− .825	− .408
B6	600	3.52	4.00	4	1.228	1.508	− .465	− .730
B7	600	3.65	4.00	4	1.215	1.477	− .733	− .301
I1	600	3.58	4.00	4	1.154	1.331	− .729	− .239
I2	600	3.02	3.00	3	1.258	1.583	.103	− .998
I3	600	3.62	4.00	4	1.092	1.192	− .675	− .057
I4	600	3.34	3.00	3	1.102	1.214	− .377	− .396
I5	600	3.35	3.00	3	1.229	1.511	− .422	− .632
I6	600	2.82	3.00	3	1.255	1.576	.243	− .903
C1	600	3.52	4.00	4	1.145	1.312	− .435	− .696
C2	600	3.34	3.00	3	1.083	1.173	− .344	− .395
C3	600	3.20	3.00	4	1.228	1.507	− .253	− .877
C4	600	3.49	4.00	4	1.170	1.369	− .456	− .584
C5	600	3.26	4.00	4	1.237	1.530	− .381	− .890
Aq1	600	3.97	4.00	5	.989	.979	− .674	− .146
Aq2	600	3.81	4.00	5	1.157	1.339	− .641	− .497
Aq3	600	3.92	4.00	4	1.053	1.108	− .945	.520
Aq4	600	3.82	4.00	4	1.062	1.127	− .710	− .156
S1	600	2.80	3.00	3	1.079	1.164	− .205	− .751
S2	600	2.84	3.00	3	1.024	1.048	− .023	− .599
S3	600	2.57	3.00	3	.944	.891	.195	− .208
S4	600	2.48	2.00	3	1.021	1.043	.311	− .354
S5	600	3.23	3.00	4	1.084	1.175	− .414	− .556
S6	600	3.02	3.00	3	1.110	1.232	− .249	− .606

## Abbreviations

AMOS: Analysis of a moment structures
AVE: Average variance extracted
CFA: Confirmatory factor analysis
CFI: Comparative fit index
CR: Composite reliability
CV: Convergent validity
EFA: Exploratory factor analysis
RMSEA: Root mean square error of approximation
SEM: Structural equation model
SRMR: Standardized root mean square residual
SPSS: Statistical Package for Social Sciences
TLI: Tucker-Lewis index
